# Exploring Spatial Inequalities in COVID-19 Mortality and Their Association With Multidimensional Poverty in Colombia: A Spatial Analysis Study

**DOI:** 10.3389/ijph.2024.1607820

**Published:** 2025-01-06

**Authors:** Claudia Birchenall-Jiménez, Wilson Giovanni Jiménez-Barbosa, Javier Riascos-Ochoa, Federico Cosenz

**Affiliations:** ^1^ Intensive Care Department, Hospital Universitario Mayor-Mederi, Bogotá, Colombia; ^2^ Escuela de Medicina y Ciencias de la Salud, Universidad del Rosario, Bogotá, Colombia; ^3^ Department of Basic Sciences and Modelling, Faculty of Natural Sciences and Engineering, Universidad Jorge Tadeo Lozano, Bogotá, Colombia; ^4^ Department of Political Sciences and International Relations, University of Palermo, Palermo, Italy

**Keywords:** multidimensional poverty index, COVID-19 mortality, Colombia, spatial distribution, socioeconomics risk factors

## Abstract

**Objectives:**

The objective is to examine spatial inequalities in COVID-19 mortality rates in Colombia in relation to the spatial distribution of multidimensional poverty.

**Methods:**

A retrospective spatial epidemiological study was conducted in Colombia from 2020 to 2022. Spatial statistics such as Moran’s I index, LISA analysis, and simultaneous autoregressive conditional (SAC) regression models were used.

**Results:**

The Moran’s I index for different years was as follows: 2020: 0.3 (p = 0.0001), 2021: 0.27 (p = 0.0001), and 2022: 0.26 (p = 0.0001). In 2020, the significant variables were low educational achievement, barriers to early childhood care, child labor, school non-attendance, informal employment, lack of health insurance, inadequate floor material, and critical overcrowding. In 2021, the significant variables were low educational achievement, critical overcrowding, inadequate excreta disposal, and lack of access to water sources. In 2022, the significant variables were school lag and inadequate excreta disposal.

**Conclusion:**

This study revealed that in Colombia, a series of socioeconomic and health factors are interconnected and contribute to COVID-19 mortality. These changes may reflect various socioeconomic, political, and environmental dynamics that shifted during the pandemic years.

## Introduction

The COVID-19 pandemic had a profound impact in Colombia and a heterogeneous effect across Latin America, exposing weaknesses and varying capacities within the region’s healthcare systems and government responses. The rapid spread of the virus, along with high infection and mortality rates, drove governments to implement a range of strategies to mitigate its effects. However, the response across Latin America was not uniform; each country adopted diverse measures according to its resources, infrastructure, and sociopolitical conditions. Argentina implemented strict quarantines from the outset, strengthening hospital capacity and coordination across government levels, while Brazil constructed field hospitals and activated its epidemiological surveillance system. In Costa Rica, public-private sector coordination was notable, providing economic assistance to the unemployed, whereas Peru expanded virus monitoring through serological testing aimed at at-risk populations. Despite these efforts, several factors hampered a coordinated response. Political tensions in Brazil and Mexico, resource shortages in Ecuador, and fragmented healthcare systems in Peru and other countries limited effective virus control. High rates of informal employment and overcrowding further hindered adherence to isolation and social distancing measures, underscoring the challenges in achieving a comprehensive and unified response across the region. Latin America thus faced not only a public health crisis but also a test of resilience and coordination, highlighting the need to strengthen healthcare systems and promote adaptive public policies for future health emergencies [1].

In Colombia, as in many countries, the COVID-19 pandemic has necessitated significant adjustments in governmental strategies, health systems, and communities [2, 3]. This occurred within a context of high inequality and territorial diversity, highlighting the increased vulnerability of impoverished communities during health and economic crises [4]. Despite economic and social advancements, Colombia continues to face challenges in addressing inequality.

In response to the global health emergency, Colombia confirmed its first COVID-19 case on 6 March 2020, and quickly expanded its diagnostic and structural capacity to contain the virus. The Unified Command Post (PMU) was established to coordinate actions with national and international organizations, and a national lockdown was implemented on March 25, followed by adjustments to restrictions as infections and ICU occupancy levels evolved. Despite four COVID-19 waves through February 2022, the Colombian healthcare system demonstrated resilience, avoiding collapse by significantly increasing hospital capacity, with a 156.2% rise in ICU beds compared to pre-pandemic levels, reaching 11,044 ICU beds and 47,640 hospital beds in 2022. Additionally, healthcare personnel were reinforced with the early graduation of around 2,500 medical students [5].

COVID-19 vaccination in Colombia began in February 2021, during a period of declining cases. Throughout the year, the country faced a third wave in May, with ICU occupancy reaching 86.3% and 22.47% of the population vaccinated. By July, the final group in the vaccination plan was prioritized, although the initial target was not met. The Delta variant emerged in the country, and cases began to decline, achieving 60% of the population vaccinated by November. At the end of 2021, with the arrival of the Omicron variant, new measures and vaccination adjustments were implemented. By January 2022, 78.7% of the population was vaccinated, and in February 2022, with 80.5% vaccinated, Colombia had accumulated 118,096 COVID-19 cases and 2,699 deaths per million [5].

The measurement of multidimensional poverty encompasses a more comprehensive perspective by considering various areas. This strategy goes beyond the simple definition of “poor” as someone with low income, recognising that poverty is a complex and multifaceted phenomenon. However, the relationship between multidimensional poverty and COVID-19 mortality has not been exhaustively investigated in low- and middle-income countries, such as Colombia. Despite the numerous studies on the Colombian Multidimensional Poverty Index (CMPI) and its association with COVID-19 mortality [6–8], the variables that have changed over time during the pandemic and their spatial relationships have not yet been identified. By analysing deprivations in areas such as education, health, childhood and youth conditions, housing, and access to basic services, multidimensional measurement allows for a more precise identification of the needs and realities of people living in poverty. Identifying these variables in different municipalities and how they were presented in various years of the pandemic facilitates the design of public policies that prioritise poverty factors, thus enabling the development of specific programs to promote regional growth. The main objective of this study is to examine spatial inequalities in COVID-19 mortality rates in Colombia in relation to the spatial distribution of multidimensional poverty. For this reason, a retrospective spatial epidemiological study was conducted, relating the Colombian multidimensional poverty index to COVID-19 mortality rates across the years 2020, 2021, and 2022.

## Methods

### Study Design and Data Collection

A retrospective spatial epidemiological study was designed, covering the period from 6 March 2020 to 31 December 2022. Demographic and mortality information was obtained from the national COVID-19 database created by the National Institute of Health of Colombia (INS) for the years 2020–2022 [9], consolidating cases diagnosed with COVID-19 infection through polymerase chain reaction (PCR) or antigen tests. Data on multidimensional poverty were sourced from the National Department of Statistics (DANE) from the 2018 population census [10].

### Measurement of Multidimensional Poverty

The CMPI index was adapted by Angulo et al. on the basis of the Alkire‒Foster method [11, 12]. This adaptation extends to 5 dimensions and 15 indicators The first dimension, educational conditions of the household, includes indicators of low educational achievement and illiteracy. The second dimension, conditions of childhood and youth, covers School non-attendance, school lag, Barriers to access for early childhood care, and child labor. The third dimension, employment, focuses on long-term unemployment and informal employment. The fourth dimension, health, includes the No Health insurance and barriers to accessing health systems. The fifth dimension, access to public services and housing conditions, considers no access to water source, inadequate excreta disposal, Inadequate floor material and inadequate wall material, and critical overcrowding [13–16]. This index was chosen because it allows for the measurement of multiple deprivations experienced by individuals simultaneously. The definition can be found in Supplementary Material S1.

### Categorisation of Municipalities

In Colombia, regulations [15–18] establish a municipal categorisation that classifies municipalities according to their population and unrestricted current revenues. The special category includes those municipalities with a population equal to or greater than 500,001 inhabitants and with current revenues exceeding 400,000 legal monthly minimum wages (SMLMs). The first category includes municipalities with a population between 100,001 and 500,000 inhabitants, with current revenues between 100,000 and 400,000 SMLM. The second category comprises municipalities with a population of 50,001–100,000 inhabitants and current revenues between 50,000 and 100,000 SMLM, whereas the third category includes those with a population of 30,001–50,000 inhabitants and current revenues between 30,000 and 50,000 SMLM. The fourth category includes municipalities with a population of 30,001–50,000 inhabitants and current revenues between 25,000 and 30,000 SMLM, and the fifth category includes those with a population of 10,001–20,000 inhabitants and current revenues between 15,000 and 25,000 SMLM. Finally, the sixth category comprises municipalities with a population equal to or less than 10,000 inhabitants and current revenues equal to or less than 15,000 SMLM, with one dollar equivalent to 3,822.05 Colombian pesos as of 31 December 2023.

### Statistical Analysis

First, the COVID-19 mortality rate was calculated by dividing the total annual deaths attributed to COVID-19 by the total projected population for the same year, according to DANE estimates, and multiplying by 100,000 to express the rate per 100,000 inhabitants. This approach uses the crude mortality rate, providing a direct and accessible measure of the impact of COVID-19 on the general population’s mortality. These data were evaluated in relation to COVID-19 mortality rates as potential explanatory factors, analysing the period from 2020 to 2022 [19].

Second, Moran’s I index statistics were employed to assess the global autocorrelation and spatial distribution of the CMPI and mortality rates (univariate analysis).

A univariate analysis of the CMPI and COVID-19 Mortality Rates in Colombia was conducted using local spatial correlation methods to identify geographic clustering patterns. In the case of the CMPI, four types of spatial clusters were identified: HH (High-High) areas, representing zones with high multidimensional poverty levels surrounded by similarly affected areas; LL (Low-Low) areas, grouping regions with low poverty levels; HL (High-Low) areas, which include zones of high poverty surrounded by areas of low poverty; and LH (Low-High) areas, which are low-poverty zones surrounded by areas with high poverty levels.

For the univariate analysis of COVID-19 Mortality Rates for the years 2020, 2021, and 2022 identify four areas: HH areas correspond to zones with high mortality rates surrounded by similarly affected regions. LL areas reflect zones with low mortality rates. HL areas encompass zones with high mortality rates surrounded by regions of low mortality, while LH areas have low mortality rates surrounded by high mortality zones. These spatial correlation patterns help identify critical hotspots and disparities in COVID-19 mortality, offering valuable insights for targeted intervention strategies.

The spatial dependence of the municipalities was represented on a map, and a colour-coding system was used to indicate the type of interaction. High-high and low-low areas reflect positive local spatial correlations identified as spatial clusters (red and blue, respectively), whereas high-low and low-high areas represent negative local spatial correlations (pink and light blue, respectively).

Third, bivariate local indicators of spatial association (LISA) clusters were generated using the CMPI and COVID-19 mortality rates to analyse the contribution of each municipality. This method enabled the identification of spatial clustering patterns by highlighting high-high (HH), low-low (LL), high-low (HL), and low-high (LH) areas through local spatial correlations. HH areas, represented in red, indicate regions with both high poverty and high mortality rates. LL areas, in blue, group regions with low levels of both poverty and mortality. HL areas, shown in pink, correspond to regions with high poverty but low mortality, while LH areas, in light blue, represent regions with low poverty rates paired with high mortality. For both spatial analyses (univariate and bivariate), Queen’s contiguity spatial lag of order 1 (immediate neighbors) was employed. The statistical significance level was set at 0.05.

Finally, the spatial simultaneous autoregressive (SAC) model was used to understand the relationships between the CMPI and its variables and COVID-19 mortality rates across different years.

Statistical analysis was conducted via R version 4.3.1 with the packages sf, spatialreg, ggplot2, and epitools.

## Results

In Colombia, during the period from 6 March 2020 to 31 December 2022, a total of 6,313,872 positive COVID-19 cases were identified. The annual distribution of cases revealed 1,746,949 cases in 2020, followed by 3,506,804 cases in 2021 and 1,060,119 cases in 2022. The mortality rates were 118.99 per 100,000 inhabitants in 2020, 200.84 per 100,000 inhabitants in 2021, and 28.99 per 100,000 inhabitants in 2022.

**FIGURE 1:**
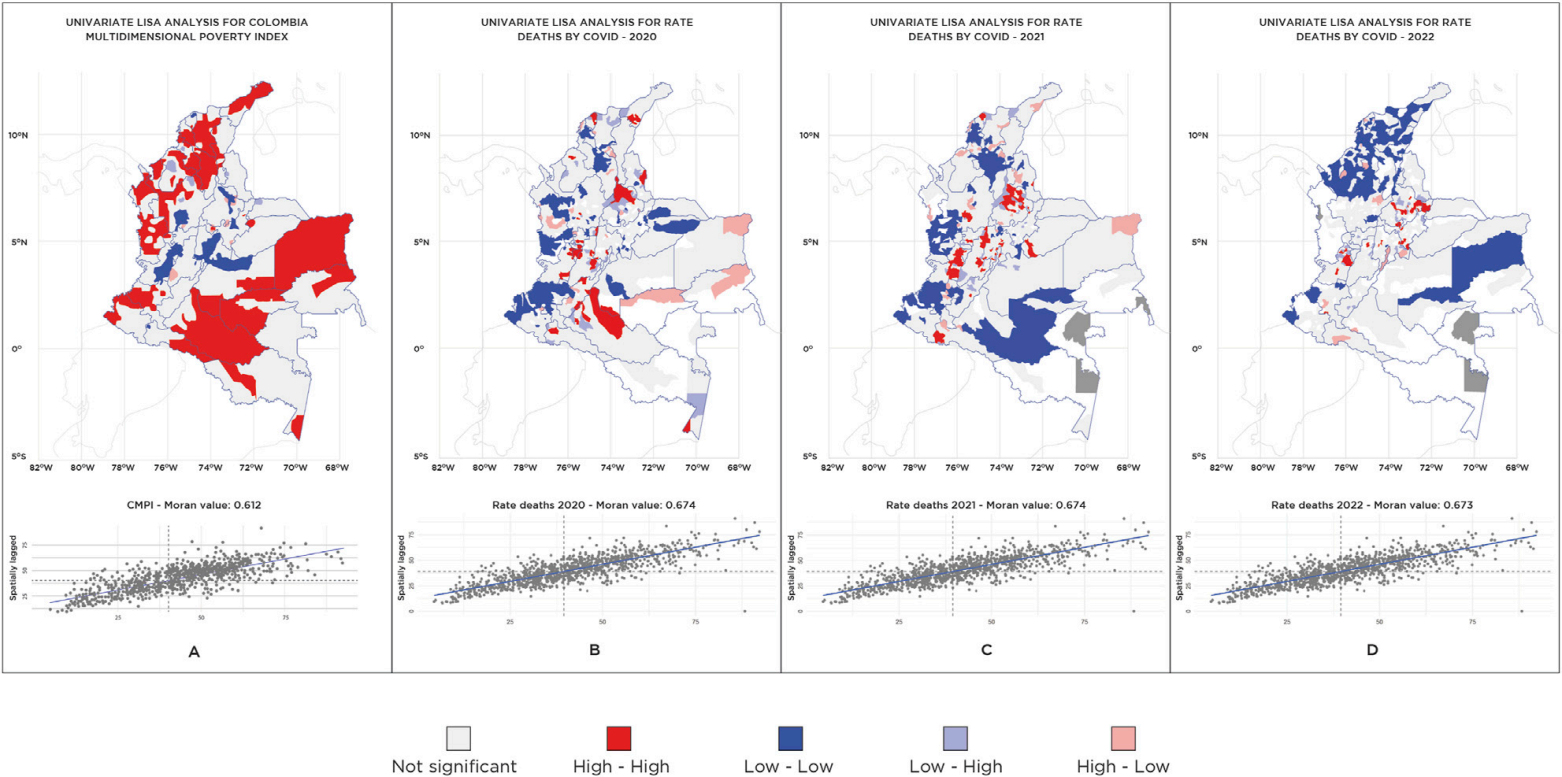
Univariate local indicator of spatial association analysis for Colombian Multidimensional Poverty Index (CMPI) (Colombia, 2018) and mortality rates (Colombia, 2020–2022).

A global spatial autocorrelation, based on a first-order queen contiguity spatial lag, was observed for the distribution of the CMPI across municipalities, with a global Moran’s I of 0.61 (p = 0.15). Similarly, global Moran’s I values for annual mortality rates were calculated as 0.674 in 2020, 0.674 in 2021, and 0.673 in 2022, all with non-significant p-values (p > 0.05), indicating an absence of significant spatial clustering for mortality rates across these years. The spatial distribution is depicted in Figure 1.

### Spatial Distribution and Autocorrelation of COVID-19 Mortality Rates Among Municipalities

The Moran’s I index for the analysed years revealed a significant spatial correlation among the COVID-19 cases in the municipalities. For 2020, the index was I = 0.3 with a p-value of 0.0001; for 2021, it was I = 0.27 with a p-value of 0.0001; and for 2022, it was I = 0.26 with a p-value of 0.0001.

In the local autocorrelation analysis via the local indicator of spatial association (LISA) test, municipalities with high COVID-19 mortality rates are found to be adjacent to those with a high multidimensional poverty index (HH). Similarly, municipalities with low COVID-19 mortality rates are contiguous with those with a low multidimensional poverty index (LL). Additionally, cases with low COVID-19 mortality rates, a high multidimensional poverty index (LH) and high COVID-19 mortality rates with a low multidimensional poverty index (HL) were identified. The categorisation of municipalities into different groups on the basis of population size, unrestricted current revenues, and spatial clustering patterns is shown in Table 1.

**TABLE 1:** Categorisation of municipalities by population size, restricted current revenues, and spatial clustering patterns. Colombia 2020–2022.

2020						
Moran Index			I = 0.3 p = 0.0001			
Municipal category	H-H	H-L	L-H	L-L	Not significance	Total
Spetial category	0	6	0	0	1	7
Category 1	1	22	0	0	11	34
Category 2	1	15	0	2	3	21
Category 3	0	13	0	4	4	21
Category 4	4	5	4	0	11	24
Category 5	3	17	1	6	14	41
Category 6	34	47	128	81	528	818
Total	43	125	133	93	572	966
2021						

Moran Index			I = 0,27 p = 0,0001			
Municipal category	H-H	H-L	L-H	L-L	Not significance	Total
Spetial category	0	6	0	0	1	7
Category 1	1	21	0	0	12	34
Category 2	1	15	0	2	3	21
Category 3	0	17	0	0	4	21
Category 4	3	4	5	1	11	24
Category 5	4	19	1	2	15	41
Category 6	25	81	156	58	621	941
Total	34	163	162	63	667	1,089
Year 2022						

Moran Index			I = 0,26 p = 0,0001			
Municipal category	H-H	H-L	L-H	L-L	Not significance	Total
Spetial category	0	6	0	0	1	7
Category 1	0	12	1	10	11	34
Category 2	0	4	1	14	2	21
Category 3	0	2	1	13	5	21
Category 4	0	2	7	1	13	23
Category 5	1	8	4	9	17	39
Category 6	8	39	91	49	460	647
Total	9	73	105	96	509	792

**FIGURE 2:**
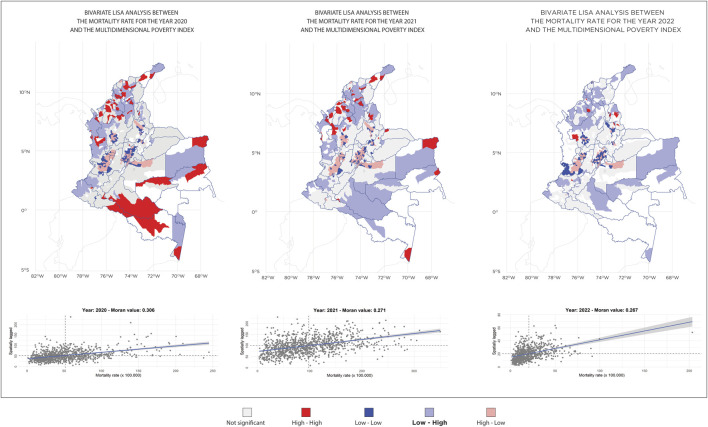
Maps of significant clusters obtained through local indicator of spatial association analysis of COVID-19 mortality rates and Colombian Multidimensional Poverty Index (CMPI). Colombia 2020–2022.

For better visualisation, the results of the LISA analysis for significant clusters are presented in Figure 2. The data by municipality and their statistical significance in the LISA analysis are provided in Supplementary Material SN2.

During the years 2020, 2021, and 2022, municipalities in categories 5 and 6 showed a predominance of high-high clusters. Although these clusters decreased over time, in 2022, the municipalities that still presented high values included Albán, Piendamó, Tunia, Pamplona, Suratá, Ocaña, Sardinata, San Marcos, Colosó, and Urrao.

In 2020, most observations were not significant, but there was a considerable number of low-high clusters, indicating a low mortality rate with a high multidimensional poverty index.

In 2021, both the high-low and low-high clusters increased, indicating greater area dispersion, with outliers distributed across various categories.

In 2022, the number of low-low clusters increased significantly, especially in Category 6.

### Spatial Modelling for Mortality Rates and CMPI Variables

The SAC model was applied for each pandemic year, and it was found that for the year 2020, the significant variables were low educational achievement (p < 0.0005, CI95%: −1.89–1.19), school non-attendance (p = 0.0079, CI95%: 0.38–2.52), barriers to early childhood care (p = 0.031, CI95%: −3.88–0.18), child labor (p = 0.0034, CI95%: −6.88–1.36), informal employment (p = 0.0053, CI95%: 0.23–1.30), no health insurance (p = 0.0077, CI95%: −0.63–0.10), inadequate floor material (p = 0.0013, CI95%: −0.54–0.13), and critical overcrowding (p=0.0015, CI95%: 0.33–1.41). The model results are presented in Table 2.

**TABLE 2:** Results of the spatial simultaneous autoregressive (SAC) model (Colombia, 2020).

Dimensions	Variable	Coefficient	Confidence interval 95%		p-value
	Rho	−0.39	−0.52	−0.26	0.0005
	(Intercept)	116.64	70.18	163.09	0.0005
Household education conditions	Low educational achievement	−1.54	−1.89	−1.19	<0.0005
	Illiteracy	−0.20	−0.75	0.35	0.47
Childhood and youth conditions	School non-attendance	1.45	0.38	2.52	0.007
	School lag	−0.60	−1.21	0.02	0.05
	Barriers to access for early childhood care	−2.03	−3.88	−0.18	0.03
	Child labor	−4.12	−6.88	−1.36	0.003
Employment (0.2)	Long-term unemployment	−0.40	−0.83	0.03	0.06
	Informal employment	0.76	0.23	1.30	0.005
Health	No Health insurance	0.79	0.04	1.54	0.03
	Barriers to access to health services	0.09	−0.42	0.60	0.73
Access to public utilities and housing conditions	No Access to water source	−0.14	−0.31	0.03	0.09
	Inadequate excreta disposal	−0.17	−0.34	0.00	0.05
	Inadequate floor material	−0.34	−0.54	−0.13	0.001
	Inadequate wall material	0.04	−0.27	0.36	0.79
	Critical overcrowding	0.87	0.33	1.41	0.001

For the year 2021, the significant variables were low educational achievement (p< 0.0005, CI95%: −2.48–−1.37), informal employment (p = 0.0267, CI95%: −0.28–2.15), no access to a water source (p = 0.0077, CI95%: −0.63–0.10), inadequate excreta disposal (p = 0.0022, CI95%: −0.70–0.15), and critical overcrowding (p = 0.0009, CI95%: 0.61–2.34). The model results are presented in Table 3.

**TABLE 3:** Results of spatial simultaneous autoregressive (SAC) model (Colombia, 2021).

Dimensions	Variable	Coefficient	Confidence interval 95%		p-value
	Rho	−0.48	−0.61	−0.35	0.0005
	(Intercept)	223.77	149.37	298.17	0.0005
Household education conditions	Low educational achievement	−1.92	−2.48	−1.37	<0.0005
	Illiteracy	−0.51	−1.35	0.33	0.23
Childhood and youth conditions	School non-attendance	1.13	−0.59	2.85	0.19
	School lag	−0.58	−1.53	0.38	0.23
	Barriers to access for early childhood care	−2.03	−4.95	0.90	0.17
	Child labor	−2.62	−6.42	1.17	0.17
Employment	Long-term unemployment	−0.38	−1.02	0.26	0.24
	Informal employment	0.96	0.11	1.81	0.02
Health	No Health insurance	0.94	−0.28	2.15	0.13
	Barriers to access to health services	−0.04	−0.82	0.74	0.92
Access to public utilities and housing conditions	No Access to water source	−0.36	−0.63	−0.10	0.007
	Inadequate excreta disposal	−0.43	−0.70	−0.15	0.002
	Inadequate floor material	−0.21	−0.53	0.10	0.18
	Inadequate wall material	−0.22	−0.76	0.32	0.42
	Critical overcrowding	1.47	0.61	2.34	0.0009

For the year 2022, the significant variables were school lag (p = 0.0450, CI95%: −0.673,933 to −0.007544) and inadequate excreta disposal (p = 0.0253, CI95%: −0.012346–0.0253). The model results are presented in Table 4.

**TABLE 4:** Results of the spatial simultaneous autoregressive (SAC) model (Colombia, 2022).

Dimensions	Variable	Coefficient	Confidence interval 95%		p-value
	Rho	0.24	0.15	0.33	<0.0005
	(Intercept)	−1.003	−21.488,353	19.48	0.92
Household education conditions	Low educational achievement	0.06	−0.08	0.21	0.42
	Illiteracy	0.02	−0.21	0.27	0.81
Childhood and youth conditions	School non-attendance	−0.39	−1.11	0.32	0.27
	School lag	−0.34	−0.67	−0.007	0.04
	Barriers to access for early childhood care	0.800,754	−0.45	2.05	0.21
	Child labor	0.07	−1.22	1.37	0.9
Employment	Long-term unemployment	0.121	−0.09	0.33	0.26
	Informal employment	0.17	−0.04	0.38	0.11
Health	No Health insurance	−0.08	−0.49	0.32	0.68
	Barriers to access to health services	0.06	−0.23	0.35	0.68
Access to public utilities and housing conditions	No Access to water source	0.03	−0.05	0.11	0.47
	Inadequate excreta disposal	−0.09	−0.18	−0.01	0.02
	Inadequate floor material	−0.06	−0.14	0.02	0.14
	Inadequate wall material	−0.08	−0.25	0.09	0.35
	Critical overcrowding	−0.09	−0.33	0.13	0.42

## Discussion

During the COVID-19 pandemic, various variables of the CMPI have been affected. In response, Colombia implemented a series of measures to contain the spread of the virus and protect its population. These measures included monetary transfer policies, social isolation, the mandatory use of face masks in public spaces, the suspension of in-person classes in educational establishments, and the promotion of mass vaccination, among others [5]. As these social interventions and vaccination campaigns were implemented, mortality rates progressively improved.

A crucial finding is that although there has been a decline in pandemic cases over the years, much work remains to be done. Municipalities in categories 5 and 6 presented the greatest number of clusters with high mortality rates and high CMPI during the studied years. These municipalities have the lowest population and the least allocation of unrestricted current revenues from the nation, along with weaker health infrastructure.

Poverty concerns not only a lack of income or resources but also a lack of access to basic services such as education, employment, healthcare, and housing. In this context, the elimination of poverty is an essential step toward sustainable development [20]. The CMPI variables that were associated with COVID-19 mortality rates from 2020 to 2022 help identify areas that can be targeted for intervention to address their deficiencies and, consequently, reduce poverty.

The analysis shows that, across all years (2020–2022), a large proportion of municipalities fell into the “Not Significant” category, indicating that these municipalities did not exhibit a clear spatial clustering pattern in COVID-19 mortality, despite the global spatial autocorrelation detected (significant Moran’s Index values across all years). This suggests that, although some autocorrelation exists in the dataset, many municipalities do not display a mortality pattern significantly related to neighboring municipalities. This could be due to unique local factors within each municipality, such as variations in healthcare infrastructure, financial constraints, local pandemic policies, areas affected by armed conflict, and mobility restrictions between municipalities.

HH areas likely represent critical hotspots that require prioritized attention to reduce vulnerability arising from the combination of high poverty levels and elevated mortality rates. In contrast, LL areas, characterized by low poverty and mortality indices, may reflect improved access to healthcare services or protective factors that have mitigated COVID-19 mortality, representing a positive aspect within the spatial correlation. For HL areas, this negative correlation pattern suggests that despite high poverty rates, these regions have maintained relatively low mortality rates, potentially indicating the effectiveness of public health interventions or specific protective factors that mitigate the impact of poverty on mortality. This presents a relevant area of investigation to identify the mechanisms that are functioning effectively within these contexts. Finally, LH areas demonstrate a negative correlation where low poverty rates are associated with high mortality rates. This finding suggests potential gaps in healthcare access or specific risk factors for COVID-19 mortality, highlighting the need for targeted interventions, as these areas do not appear to benefit from their favorable economic conditions in terms of reduced mortality.

The predominance of municipalities in spatial divergence categories (high-low and low-high) in 2020, totaling 258 municipalities, highlights an unequal distribution of COVID-19 mortality among them. This pattern suggests that municipalities with high mortality rates were surrounded by others with low rates, and *vice versa*. The observed divergence reflects how local disparities in resources and pandemic response influenced the distribution of mortality, underscoring inequalities in the impact of COVID-19 across different municipal contexts.

In 2020, educational barriers, child labour, and school non-attendance were prominent, alongside issues of informal employment and health, such as a no health insurance and inadequate housing materials. In 2021, there was continuity in problems of informal employment and health, but there was an increase in the lack of access to essential public services such as potable water and proper excreta disposal. For 2022, school lag and inadequate excreta disposal were significant issues Supplementary Material S3.

These changes may reflect various socioeconomic, political, and environmental dynamics. The persistence of informal employment suggests ongoing challenges in formalising the labour market. Improvements or deteriorations in health and housing may be related to government policies, changes in access to public resources, and the evolution of crises such as the COVID-19 pandemic, which may have exacerbated some of these conditions, especially in terms of overcrowding and access to basic services.

With respect to each of the variables, this study identified low educational achievement, school lag, and school non-attendance. Low educational achievement, characterised by an average of fewer than 9 years of education in the household, has emerged as a significant risk factor. This phenomenon is closely related to employment in essential, poorly paid jobs, where individuals may face greater exposure to the virus and limited medical care, potentially making them vectors of contagion. This, in turn, affects family members living with them, who may be part of more vulnerable populations, such as the elderly and patients with chronic diseases [21, 22].

Moreover, school lag refers to the situation in which children and adolescents aged 13–17 do not achieve the educational level required for their age group, as stipulated by national educational policies. This phenomenon can be largely attributed to the interruption of in-person classes at all educational levels during the pandemic, which has created a gap in knowledge acquisition. The COVID-19 pandemic has had a significant impact on education in Colombia, as demonstrated by the school absenteeism rate. In 2020, school absenteeism peaked at 16.4%, probably due to interruptions in in-person education and the challenges associated with transitioning to remote learning. However, a notable decrease in school absenteeism was observed in the following years, dropping to 5.5% in 2021 and 2.3% in 2022 [10]. These figures indicate a gradual recovery in school attendance as schools began to reopen and measures were implemented to support remote education [23]. Nevertheless, the pandemic has exposed and, in many cases, exacerbated existing inequalities in the educational system.

The lack of comprehensive access to early childhood care is related to the absence of simultaneous health, nutrition, and early education services. This study revealed an association between this domain and COVID-19 mortality. Disparities in access are due to familial, community, environmental, and political factors [24, 25] and are exacerbated by economic, cultural, and knowledge barriers [26]. World Bank strategies and studies on women’s economic empowerment suggest that improving childcare can have positive effects on early childhood development and gender equity [27, 28].

Child labour is defined as the exploitation of children through any form of work that deprives them of their childhood, interferes with their ability to attend school regularly, and is detrimental to their development. Although there has been a gradual reduction in the child labor rate in Colombia, from 5% in the last quarter of 2020 to 4.8% in 2021 and 3.4% in 2022 [29], the figure remains concerning, especially in populated centers and dispersed rural areas, where it reaches 55.6% [30]. Importantly, poverty, intensified by the COVID-19 pandemic, can aggravate this phenomenon. Child labour not only deprives children of their right to education by forcing them to leave school or balance it with long working hours but also affects their physical, mental, and social development [31].

In Colombia, a significant portion of the population works in the informal sector, facing a lack of social protection and pension rights. The informality rate remains high, exceeding 50% of total employment [32]. This disparity translates into greater vulnerability to illness due to their economic necessity, which has led to noncompliance with isolation measures designed to prevent the spread of the virus [33–35].

The lack of healthcare coverage restricts access to medical and preventive services, thereby increasing the risk of complications and untreated illnesses. This scenario highlights socioeconomic inequalities, exacerbating health disparities. According to the DANE figures, the prevalence of health insurance was 10.8% in 2020, 10.1% in 2021, and 8.4% in 2022 [10].

Research has indicated that housing conditions, such as critical overcrowding and inadequate flooring material, had a significant impact on COVID-19 mortality from 2020 to 2021 [36, 37]. Urban planning and public policies must ensure that housing infrastructure meets basic standards of habitability and health.

Additionally, a lack of access to improved water sources and inadequate excreta disposal were associated with mortality in 2021 and 2022. Water supply coverage in urban areas reached 97.9%, whereas in rural areas, it reached 71.54%. Inadequate excreta disposal remains a significant variable [38]. With respect to sewerage, 92.85% of urban areas and 73.88% of rural areas have coverage [39]. Moreover, the construction of aqueducts and adequate excreta disposal in Colombia has proven effective in preventing pandemics [40]. Despite improvements in these indicators over recent years, these variables are associated with COVID-19 mortality rates.

The limitations of this study include the inconsistent or incomplete quality and availability of data in some municipalities. The population was not disaggregated by gender owing to the need to prioritise a generalised analysis that allows for addressing broader trends and issues, ensuring that all population dimensions are included to obtain a holistic view of the pandemic’s impacts. The lack of detailed longitudinal data and the variability in local responses to the pandemic also present significant challenges. Confounding factors, such as population mobility and cultural practices, could influence the results. Additionally, the study does not incorporate variables related to health services, which could have a significant impact on COVID-19 mortality.

### Conclusion

This study revealed that in Colombia, a series of socioeconomic and health factors are interconnected and contribute to COVID-19 mortality. These changes may reflect various socioeconomic, political, and environmental dynamics that shifted during the pandemic years. Low educational achievement, school absenteeism, inadequate childcare, child labour, informal employment, lack of health insurance coverage, and housing challenges—including limited access to public water and sewer services, overcrowding, and poor-quality materials—were linked to a higher risk of mortality among family members.

This work underscores the importance of understanding socioeconomic and spatial dynamics as essential elements for defining, addressing, and intervening in problems associated with emergencies such as the COVID-19 pandemic. By doing so, it is possible to formulate and implement public policies that can prevent, mitigate, and overcome these issues, thereby minimising the impact on the wellbeing and lives of affected individuals.
