# Supplementary material for: Exploring Spatial Inequalities in COVID-19 Mortality and Their Association With Multidimensional Poverty in Colombia: A Spatial Analysis Study
Source: Int J Public Health. 2025 Jan 6;69:1607820. doi: 10.3389/ijph.2024.1607820 (PMC11742940; doi:10.3389/ijph.2024.1607820)
Supplement: Supplementary file 2 [file Table1.DOCX]

Supplement 1

Table 1. Dimensions and variables of the CMPI (11)

| **DIMENSIONS** | **VARIABLE** | | **CUT OFF POINT** |
| --- | --- | --- | --- |
|  | **VARIABLE** | **INDICATOR** |  |
| Household education conditions (0.2) | Low educational achievement (0.1) | Average education level for people 15 and older living in a household | 9 years |
|  | Illiteracy (0.1) | Percentage of people living in a household 15 and older who know how to read and write | 100% |
| Childhood and youth conditions (0.2) | School non-attendance (0.05) | Percentage of children between the ages of 6 and 16 in the household that attend school | 100% |
|  | School lag (0.05) | Percentage of children and youths (7–17 years old) within the household that are not suffering from school lag (according to the national norm) | 100% |
|  | Barriers to access for early childhood care (0.05) | Percentage of children between the ages of 0 and 5 in the household who simultaneously have access to health, nutrition and education | 100% |
|  | Child labor, (0.05) | Percentage of children between 12 and 17 years old in the household that are not working | 100% |
| Employment (0.2) | Long-term unemployment (0.1) | Percentage of a household´s EAP that is not facing long-term unemployment (more than 12 months) | 100% |
|  | Informal employment (0.1) | Percentage of a household´s EAP that is employed and affiliated with a pension fund (formality proxy) | 100% |
| Health (0.2) | No Health insurance (0.1) | Percentage of household members over the age of 5 that are insured by the Social Security Health System | 100% |
|  | Barriers to access to health services (0.1) | Percentage of people within the household that has access to a health institution in case of need | 100% |
| Access to public utilities and housing conditions (0.2) | No Access to water source (0.04) | Urban households are considered deprived if lacking public water system. Rural households are considered deprived when the water used for the preparation of food is obtained from wells, rainwater, spring source, water tank, water carrier or other sources. | 1 |
|  | Inadequate excreta disposal (0.04) | Urban households are considered deprived if they lack a public sewer system. Rural households are considered deprived if they use a toilet without a sewer connection, a latrine or simply do not have a sewage system. | 1 |
|  | Inadequate floor material (0.04) | Households with dirt floors are considered deprived. | 1 |
|  | Inadequate wall material (0.04) | An urban household is considered deprived when the exterior walls are built of untreated wood, boards, planks, guadua or other vegetation, zinc, cloth, cardboard, waste material or when no exterior walls exist. A rural household is considered deprived when exterior walls are built of guadua or other vegetation, zinc, cloth, cardboard, waste materials or if no exterior walls exist. | 1 |
|  | No critical overcrowding (0.04) | Number of people sleeping per room, excluding the kitchen, bathroom and garage | Urban: 3 or more people per room Rural: More than 3 people per room |
