# Supplementary figures and images for: Exploring Spatial Inequalities in COVID-19 Mortality and Their Association With Multidimensional Poverty in Colombia: A Spatial Analysis Study
Source: Int J Public Health. 2025 Jan 6;69:1607820. doi: 10.3389/ijph.2024.1607820 (PMC11742940; doi:10.3389/ijph.2024.1607820)

Supplement 3

CMPI Variables Over Time.


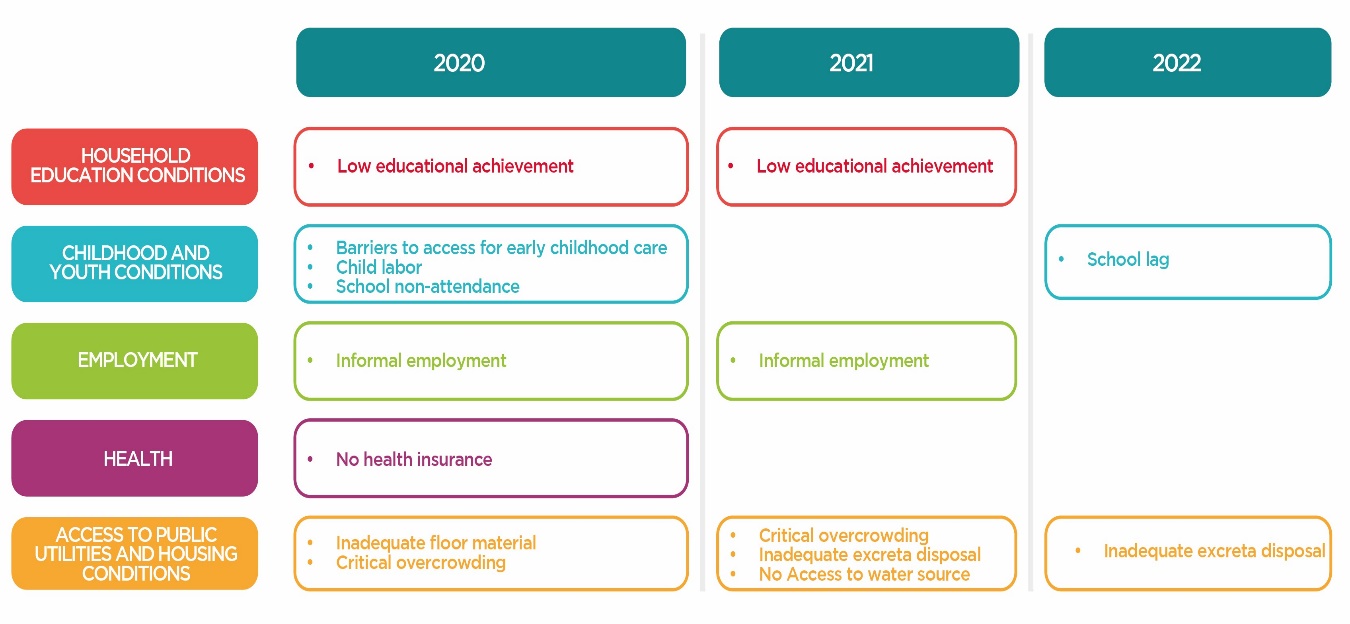

Supplement: Supplementary file 3 [file Table3.DOCX]
